# Supplementary material for: Toolkit for Population Health Initiatives Around the Globe Related to Collaborative Comprehensive Medication Management for Children and Youth
Source: Children (Basel). 2019 Apr 8;6(4):57. doi: 10.3390/children6040057 (PMC6518279; doi:10.3390/children6040057)
Supplement: Supplementary file 1 [file children-06-00057-s001.pdf]

Table 1. Evidence of expectation for key clinical pharmacist practice activities in each continent [8].

Expected activity in North American clinical practice	Africa	Asia	Europe	Oceania	South America
Assesses the status of the patient's health problems and determine whether the prescribed medications are optimally meeting the patient's needs and goals of care	+	+	+++	+++	+
Evaluates the appropriateness and effectiveness of the patient's medications	+	+	++	+++	+
Recognizes untreated health problems that could be improved or resolved with appropriate medication therapy			++	+++	
Follows the patient's progress to determine the effects of the patient's medications on his or her health			++	+++	
Consults with the patient's physicians and other health care providers in selecting the medication therapy that best meets the patient's needs and contributes effectively to the overall therapy goals	+	+	+++	+++	+
Advises the patient on how to best take his or her medications	+++	+++	+++	+++	+++
Supports the health care team's efforts to educate the patient on other important steps to improve or maintain health, such as exercise, diet, and preventive steps like immunization			++	+++	
Refers the patient to his or her physician or other health professionals to address specific health, wellness, or social services concerns as they arise			+++	+++	

Key: + = developing expectation on the continent; ++ = published expectation on the continent; +++ = established expectation on the continent
